# New Zirconium Diboride Polymorphs—First-Principles Calculations

**DOI:** 10.3390/ma13133022

**Published:** 2020-07-06

**Authors:** Marcin Maździarz, Mościcki Tomasz

**Affiliations:** Institute of Fundamental Technological Research Polish Academy of Sciences, 02-106 Warsaw, Poland; tmosc@ippt.pan.pl

**Keywords:** zirconium diboride, ab initio calculations, mechanical properties, elastic properties, phonons

## Abstract

Two new hypothetical zirconium diboride (ZrB${}_{2}$) polymorphs: (*hP6*-P6${}_{3}$/mmc-space group, no. 194) and (*oP6*-Pmmn-space group, no. 59), were thoroughly studied under the first-principles density functional theory calculations from the structural, mechanical and thermodynamic properties point of view. The proposed phases are thermodynamically stable (negative formation enthalpy). Studies of mechanical properties indicate that new polymorphs are less hard than the known phase (*hP3*-P6/mmm-space group, no. 191) and are not brittle. Analysis of phonon band structure and density of states (DOS) also show that the phonon modes have positive frequencies everywhere and the new ZrB${}_{2}$ phases are not only mechanically but also dynamically stable. The estimated acoustic Debye temperature, $\Theta_{D}$, for the two new proposed ZrB${}_{2}$ phases is about 760 K. The thermodynamic properties such as internal energy, free energy, entropy and constant-volume specific heat are also presented.

## 1. Introduction

During latest years, the transition metal borides have attracted attention among materials researchers due to the combination of their outstanding physical properties such as electric and thermal conductivity comparable with metals, low compressibility, high shear strength and exceptionally high hardness [1,2]. Even in the form of thin films, they possess extraordinary properties such as very high hardness with increased flexibility, great thermal properties and very good corrosion and wear resistance [3,4,5]. Among borides, zirconium diboride (ZrB${}_{2}$) deserves special attention. ZrB${}_{2}$, with melting temperature 3245 ${}^{\circ }$C [6], is a member of a family of materials known as ultra-high temperature ceramics (UHTCs). In addition to high melting temperatures, ZrB${}_{2}$ has a unique combination of chemical stability, high electrical and thermal conductivities and resistance to erosion and corrosion that makes its suitable for the extreme chemical and thermal environments associated with, for example, hypersonic flight, atmospheric re-entry and rocket propulsion [7]. According to the Zr–B phase diagram [8,9] there are three phases, namely, ZrB, ZrB${}_{2}$, and ZrB${}_{12}$, which have been reported and widely studied for this system. Theoretical investigations show that ZrB can create different crystallographic structures. The basic phase is NaCl-type face-centered cubic ZrB (*Fm-3m*-space group, no. 225) with lattice constant a = 4.900 Å [10]. Furthermore, ZrB also can crystallize in a FeB-type structure with a primitive orthorhombic (*Pnma*) crystal structure [9,11], CrB-type orthorhombic structure with *Cmcm*-space group [11,12] and hexagonal *Pmmm* [13]. A literature review shows that ZrB${}_{12}$ is only stable in one structure type. The cubic LuB${}_{12}$ structure (*Fm3m*-space group, no. 225) with lattice constant a = 7.4085 Å was studied theoretically and experimentally for example in Reference [14]. They compared electric-field gradient measurements at the B sites and first-principles calculations in order to analyse the chemical bonding properties. Both experimental and theoretical results were in good agreement. In Reference [15], however, the mechanical properties of ZrB${}_{12}$ were calculated. The Vickers hardness of zirconium boride was 32.9 GPa, which is in good agreement with other theoretical results [14,16,17].

In contrast to ZrB${}_{12}$, depending on the calculation method, different hardness values have been determined for zirconium diboride ZrB${}_{2}$. The calculated values of hardness range from 12.82–55.53 GPa [18], whereas experimentally measured hardness reached 23 ± 0.9 GPa [6,19]. All mentioned structures were assigned as ZrB${}_{2}$ with the crystal hexagonal structure of AlB${}_{2}$-type with the *P6/mmm*-space group, no. 191. Such large differences in the values of the analysed properties may, however, come not only from differences in calculation methods but also from the possibility of the existence of other stable forms of ZrB${}_{2}$, which may form nanocomposites of different polymorphs of ZrB${}_{2}$. A similar conclusion about the possibility of the existence of other ZrB${}_{2}$ crystal types can be drawn on the basis of other studies on possible forms of transition metal diborides, for example, WB${}_{2}$ [20,21] or ReB${}_{2}$ [22,23]. For comparison, in Reference [21] the authors proposed six different phases of WB${}_{2}$. In the case of ZrB${}_{2}$, it is hard to find such a study.

It should also be noted that in addition to the phases appearing in the Zr-B equilibrium diagram [8,24], other zirconium and boron compounds have been theoretically determined. There are hypothetical Zr-B phases such as: Zr${}_{3}$B${}_{4}$, Zr${}_{2}$B${}_{3}$, Zr${}_{3}$B${}_{2}$ [16], ZrB${}_{3}$ [25], ZrB${}_{4}$ [26] and ZrB${}_{6}$ [13]. All polymorphs are both mechanically and dynamically stable but have not been confirmed experimentally yet. In this work, structural, mechanical and thermodynamic properties of stable ZrB${}_{2}$ polymorphs from density functional calculations will be studied.

## 2. Computational Methods

First-principle calculations based on density functional theory (DFT) [27,28] within the pseudopotential plane-wave approximation (PP-PW) implemented in ABINIT [29,30] code were performed in this work. Projector augmented-wave formulation (PAW) pseudopotentials [31] were used to describe the interactions of ionic core and non-valence electrons.

To enhance the confidence of the calculations as an exchange-correlation (XC) functional, three approximations were used: local density approximation (LDA) [32,33], classical Perdew-Burke-Ernzerhof (PBE) generalised gradient approximation (GGA) [34] and modified Perdew-Burke-Ernzerhof GGA for solids (PBEsol) [35]. There is a strong view that the PBEsol is the overall best performing XC functional for identifying the structure and elastic properties [36,37,38].

Projector augmented wave method (PAW) pseudopotentials used for LDA and PBE XC functionals were taken from PseudoDojo project [39]. PAW pseudopotentials for PBEsol exchange-correlation functional [35] were generated using ATOMPAW software [40] and a library of exchange-correlation functionals for density functional theory, LibXC [41].

All calculations were made by tuning the precision of the calculations, which was done by automatically setting the variables at *accuracy* level 4 (*accuracy* = 4 corresponds to the default tuning of ABINIT). The *cut-off* energy consistent with PAW pseudopotentials of the plane-wave basis set was 15 Ha with $4d^{2}5s^{2}$ valence electrons for Zr and $2s^{2}2p^{1}$ valence electrons for B. K-PoinTs grids were generated with *kptrlen* = 30.0 (grids that give a length of smallest vector LARGER than *kptrlen*). Metallic occupation of levels with the Fermi-Dirac smearing occupation scheme and *tsmear* (Ha) = 0.02 was used in all ABINIT calculations.

### 2.1. Optimization of Structures

As mentioned earlier, tungsten diboride [20] and rhenium diboride [23] crystallise in various space groups. Searching for new structures of ZrB${}_{2}$, we started with basic cells of *hP6*-P6${}_{3}$/mmc-WB${}_{2}$ and *oP6*-Pmmn-WB${}_{2}$ and replaced tungsten atoms with zirconium atoms, wherein the designations mean: Pearson symbol, Space group and Chemical formula [20]. Then, all structures were relaxed by using the Broyden-Fletcher-Goldfarb-Shanno minimisation scheme (BFGS) with full optimisation of cell geometry and atomic coordinates. Maximal stress tolerance (GPa) was set to 1 × 10${}^{-4}$.

### 2.2. Formation Enthalpy and Cohesive Energy

The formation enthalpy and cohesive energy were determined as follows [23,42]:

(1) $$\begin{array}{c} {▵}_{f}H\left({ZrB}_{2}\right)=E_{coh}\left({ZrB}_{2}\right)-E_{coh}\left(Zr\right)-2E_{coh}\left(B\right), \end{array}$$

(2) $$\begin{array}{c} E_{coh}\left({ZrB}_{2}\right)=E_{total}\left({ZrB}_{2}\right)-E_{iso}\left(Zr\right)-2E_{iso}\left(B\right), \end{array}$$

where ${▵}_{f}H\left({ZrB}_{2}\right)$ is the formation enthalpy of the ZrB${}_{2}$; $E_{coh}\left(ZrB_{2}\right)$ is the cohesive energy of the ZrB${}_{2}$; $E_{coh}\left(Zr\right)$ is the cohesive energy of Zr; $E_{coh}\left(B\right)$ is the cohesive energy of B; $E_{tot}\left(ZrB_{2}\right)$ is the total energy of the ZrB${}_{2}$; $E_{iso}\left(Zr\right)$ is the total energy of a Zr atom and $E_{iso}\left(B\right)$ is the total energy of a B atom.

The cohesive energy of the $E_{coh}\left(Zr\right)$ is calculated, taking into account stoichiometry, as the total energy difference of *zirconium* crystal (*hP2*-P6/mmc-space group, no.194) and single Zr atom in the box, whereas $E_{coh}\left(B\right)$ is the total energy difference of α-boron crystal (*hR12*-R-3m-space group, no. 166) and single B atom in a sufficiently large box [23].

### 2.3. Mechanical Properties Calculations

The theoretical ground state elastic constants $C_{ij}$ of all structures were established with the metric tensor formulation of strain in density functional perturbation theory (DFPT) [43]. Isotropised bulk modulus ***B***, shear modulus ***G***, Young’s modulus *E* and Poisson’s ratio ν were estimated by means of a *Voigt–Reuss–Hill* average [44,45].

In order to verify the elastic stability of all the structures, positive definiteness of the stiffness tensor was checked [46] by calculating Kelvin moduli, that is, eigenvalues of stiffness tensor represented in *second-rank tensor* notation [47].

Hardness of ZrB${}_{2}$ polymorphs in the present paper was calculated with the use of semi-empirical relation proposed in Reference [48]. The equation is defined as follow:

(3) $$\begin{array}{c} H_{v}=0.92{\left(G/B\right)}^{1.137}G^{0.708}. \end{array}$$

*G/B* ratio appearing in the above formula named Pugh’s modulus ratio [49] is commonly used as a universal ductile-to-brittle criterion.

### 2.4. Phonon and Thermodynamic Properties Calculations

To calculate phonons, DFPT was utilised [29,30]. The phonon dispersion curves [50] of the analysed structures were then used to determine their dynamical stability [46,51], complementary to elastic stability. Acoustic Debye temperature was calculated from the phonon densities of states (DOS).

Using the calculated phonons under the harmonic approximation, that is, in the range up to Debye temperature, thermal quantities: phonon internal energy, free energy, entropy and constant-volume heat capacity as a function of the temperature were determined [24,52].

## 3. Results

Using the approach described in Section 2.1, the first step in our calculations was the geometry optimisation of the two new hypothetical (ZrB${}_{2}$) polymorphs—(*hP6*-P6${}_{3}$/mmc-space group, no.194) and (*oP6*-Pmmn-space group, no. 59), and the formerly known polymorph (*hP3*-P6/mmm-space group, no. 191).

### 3.1. Structural Properties

**Figure 1:**
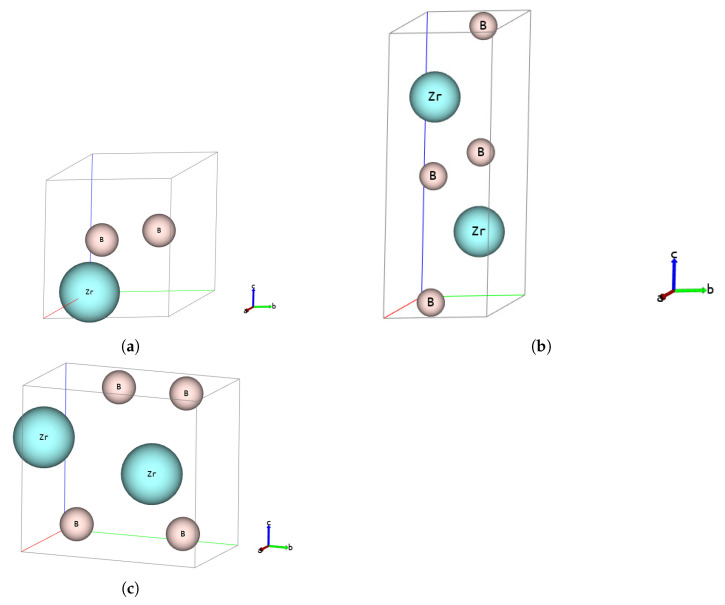
ZB${}_{2}$-Basic cells: (**a**) *hP3*-P6/mmm, (**b**) *hP6*-P6${}_{3}$/mmc and (**c**) *oP6*-Pmmn.

The basic cells for all three analysed polymorphs are depicted in Figure 1, whereas the crystallographic data, calculated with three different XC functionals (LDA, PBE and PBEsol), are stored in crystallographic information files (CIFs) in Supplementary Materials.

Determined lattice parameters, formation enthalpy and cohesive energy for known *hP3*-P6/mmm polymorph (Figure 1a) are comparable to those of other authors [18] (see Table 1). We treat this as a verification of the correctness of the methodology used.

The first new hypothetical phase, *hP6*-P6${}_{3}$/mmc (Figure 1b), also crystallises in the hexagonal system but has 6 atoms in the cell, whereas the second new hypothetical phase, *oP6*-Pmmn (Figure 1c), crystallises in the orthorhombic system and also has 6 atoms in the cell. There is little sense to compare lattice parameters for phases in different systems, but it is worth comparing the formation enthalpy and the cohesive energy. It can be seen that the formation enthalpy, ${▵}_{f}H$, for new phases is significantly lower than for the known *hP3*-P6/mmm phase and comparable between the new phases (see Table 1). The calculated cohesive energy, $E_{c}$, is a little higher than for the known *hP3*-P6/mmm phase and again comparable between the two new phases. These two facts suggest that the new phases are comparably thermodynamically stable but less stable than the known *hP3*-P6/mmm phase.

### 3.2. Mechanical Properties

Computed elastic constants, Kelvin moduli, isotropised bulk, shear and Young’s modulus, Poisson’s ratio, *G/B* Pugh’s modulus ratio, Debye temperature and estimated hardness of all analysed zirconium diboride structures are listed in Table 1. For known *hP3*-P6/mmm phase (Figure 1a), these quantities are comparable to those of other authors [18], which is a further verification of the validity of the methodology used.

Analysing the data received, it can be concluded that the new phases have lower mechanical parameters than the known *hP3*-P6/mmm phase, except for the Poisson’s ratio. Both new phases have a similar isotropised bulk modulus of about 170 GPa, but the shear modulus for the *hP6*-P6${}_{3}$/mmc phase is much lower and is only about 40 GPa. The consequence of this is that the hardness of the *hP6*-P6${}_{3}$/mmc phase is only 2 GPa, while for the *oP6*-Pmmn phase it is about 14 GPa. A high *G/B* Pugh’s modulus ratio would correspond to a more brittle than ductile character of material. The critical value, separating ductile from brittle materials, is approximately 0.571 [49,53]. It can be seen that the known *hP3*-P6/mmm phase is brittle, the *hP6*-P6${}_{3}$/mmc phase is ductile and the *oP6*-Pmmn phase is somehow between brittle and ductile.

All analysed structures have a positive definite stiffness tensor and positive Kelvin moduli, that is, eigenvalues of stiffness tensor represented in *second-rank tensor* notation, so there are mechanically stable (see Table 1).

### 3.3. Phonon and Thermodynamic Properties

**Figure 2:**
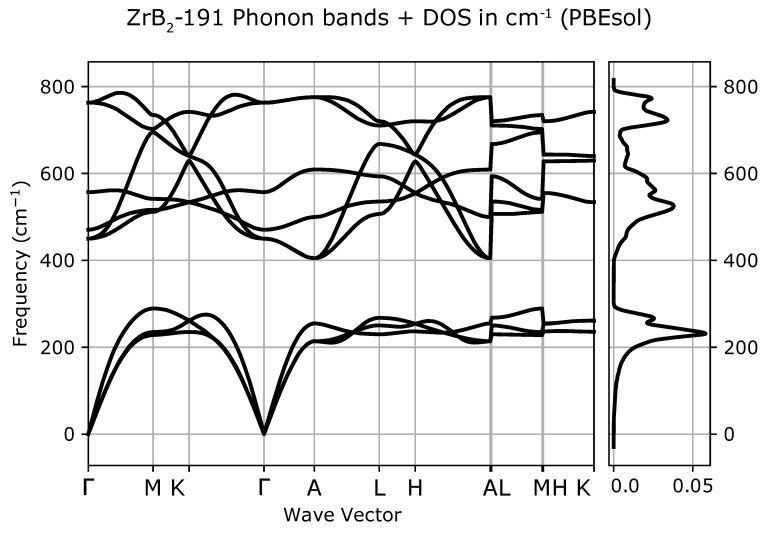
ZrB${}_{2}$(*hP3*-P6/mmm-space group, no. 191)-phonon band structure and densities of states (DOS).

**Figure 3:**
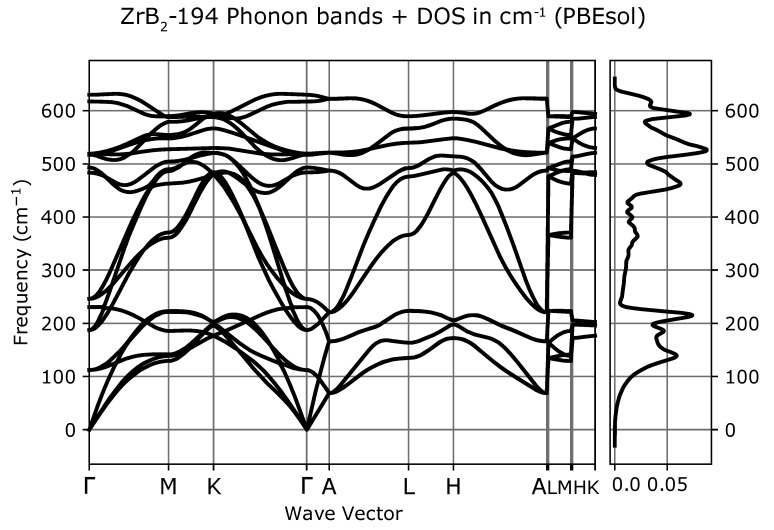
ZrB${}_{2}$(*hP6*-P6${}_{3}$/mmc-space group, no. 194)-phonon band structure and DOS.

**Figure 4:**
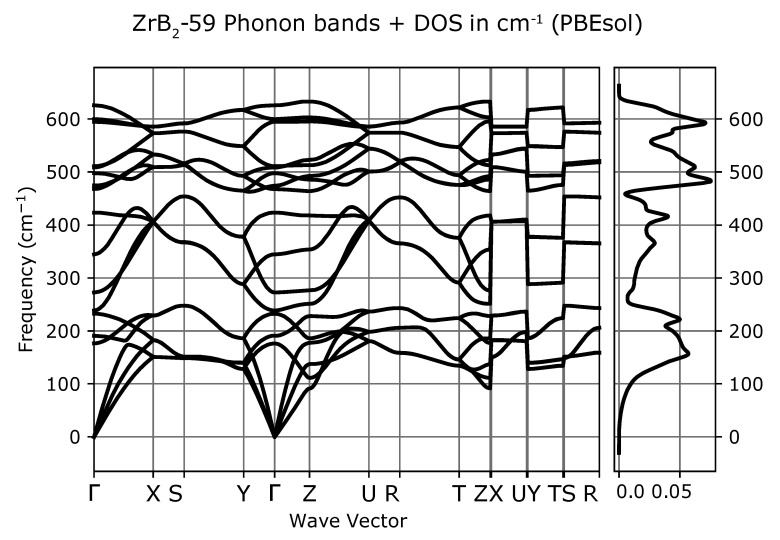
ZrB${}_{2}$(*oP6*-P6/mmm-space group, no.59)-phonon band structure and DOS.

**Figure 5:**
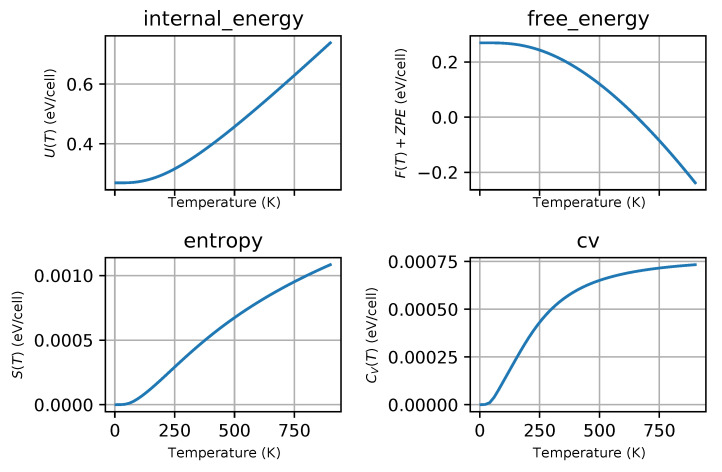
ZrB${}_{2}$(*hP3*-P6/mmm-space group, no. 191)-thermodynamic properties: internal energy, free energy, entropy and constant-volume specific heat.

**Figure 6:**
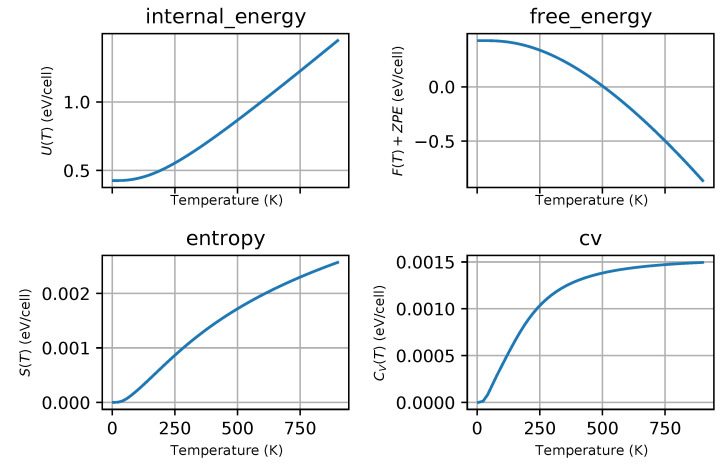
ZrB${}_{2}$(*hP6*-P6${}_{3}$/mmc-space group, no.194)-thermodynamic properties: internal energy, free energy, entropy and constant-volume specific heat.

**Figure 7:**
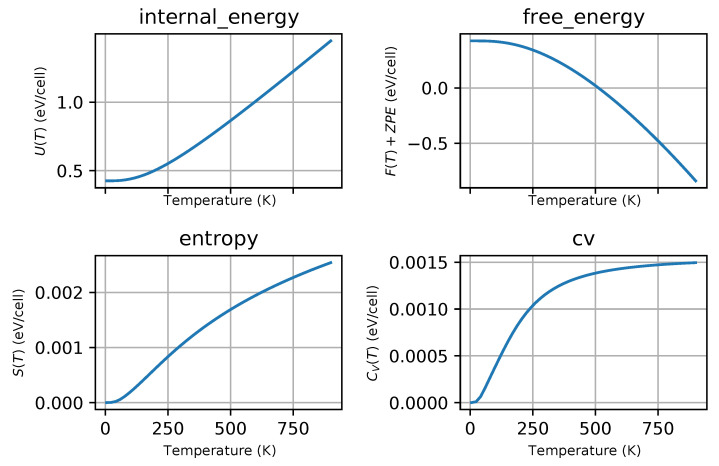
ZrB${}_{2}$(*oP6*-P6/mmm-space group, no.59)-thermodynamic properties: internal energy, free energy, entropy and constant-volume specific heat.

Phonon dispersion curves along the high symmetry q-points [50] and phonon densities of states (DOS) calculated with the use of PBEsol exchange-correlation (XC) functional for known *hP3*-P6/mmm phase, Figure 1a), are depicted in Figure 2, for the phase *hP6*-P6${}_{3}$/mmc, Figure 1b), in Figure 3 and for the phase *oP6*-Pmmn, Figure 1c), in Figure 4, respectively. Phonon results for all exchange-correlation (XC) functionals are stored in Supplementary Materials. Analysis of the calculated curves allows us to state that phonon modes everywhere have positive frequencies and the new ZrB${}_{2}$ phases are not only mechanically but also dynamically stable. The estimated acoustic Debye temperature $\Theta_{D}$ for the two new proposed ZrB${}_{2}$ phases is about 760 K and is about 200 K lower than that for the known *hP3*-P6/mmm phase and it is consistent with the mechanical properties, see Table 1. Results for thermodynamic properties up to 760 K for the three zirconium diboride polymorphs calculated with the use of PBEsol exchange-correlation (XC) functional, that is, phonon internal energy, free energy, entropy, constant-volume heat capacity are depicted in Figure 5, Figure 6 and Figure 7 and it can be seen that are very similar for the two new proposed ZrB${}_{2}$ polymorphs. This additional fact suggests again that the new phases are comparably thermodynamically stable up to Debye temperature $\Theta_{D}$, but less stable than the known *hP3*-P6/mmm phase.

## 4. Conclusions

In the present paper, extensive analysis of two new hypothetical and one previously known zirconium diboride (ZrB${}_{2}$) polymorphs within the framework of density functional theory from the structural, mechanical and thermodynamic properties point of view was performed. We can conclude that:

- two new hypothetical zirconium diboride (ZrB${}_{2}$) polymorphs: (*hP6*-P6${}_{3}$/mmc-space group, no. 194) and (*oP6*-Pmmn-space group, no. 59) are mechanically and dynamically stable;
- these phases are comparably thermodynamically stable but less stable than the known *hP3*-P6/mmm phase;
- *hP6*-P6${}_{3}$/mmc phase is ductile and *oP6*-Pmmn phase is intermediate between brittle and ductile;
- both new phases have a lower hardness than the known *hP3*-P6/mmm phase.

We hope that results relating to new hypothetical zirconium diboride (ZrB${}_{2}$) polymorphs will be confirmed by other calculations, as well as through experiments. Knowledge about these new phases can be very useful when doping metal borides with zirconium.

## Figures and Tables

**Table 1:** Lattice parameters (Å); formation enthalpy ${▵}_{f}H$ (eV/Atom); cohesive energy $E_{c}$ (eV/Atom); elastic constants  $C_{ij}$ (GPa); Kelvin moduli $K_{i}$ (GPa); bulk modulus *B* (GPa); shear modulus *G* (GPa); Young’s modulus *E* (GPa); Poisson’s ratio ν; G/B Pugh’s modulus ratio; Debye temperature $\Theta_{D}$ (K); hardness $H_{v}$ (GPa), of ZrB${}_{2}$ phases: ZrB${}_{2}$ (*hP3*-P6/mmm-space group, no. 191; *hP6*-P6${}_{3}$/mmc-space group, no.194; and *oP6*-P6/mmm-space group, no.59). Experimental and calculated data for *hP3*-P6/mmm phase are taken from Reference [18].

Phase	*hP3*-P6/mmm-No.191					*hP6*-P6${}_{3}$/mmc-No. 194			*oP6*-Pmmn-No. 59		
Source	Exp.	Calc.	LDA	PBE	PBEsol	LDA	PBE	PBEsol	LDA	PBE	PBEsol
*a*	3.165–3.169	3.127–3.197	3.135	3.173	3.156	3.025	3.076	3.050	3.057	3.100	3.071
*b*									4.931	5.029	4.981
*c*	3.523—3.547	3.490–3.561	3.477	3.527	3.495	8.515	8.624	8.565	4.541	4.604	4.578
$-{▵}_{f}H$	1.113	0.985–1.099	1.145	1.078	1.141	0.211	0.195	0.218	0.164	0.158	0.178
$-E_{c}$		5.67–8.648	8.769	8.072	8.411	7.834	7.187	7.488	7.187	7.150	7.448
$C_{11}$	581	551–606	618	591	597	224	214	217	333	325	336
$C_{22}$	581	551–606	618	591	597	224	214	217	331	316	334
$C_{33}$	445	436–482	477	481	456	495	447	479	436	380	439
$C_{44}$	240	240–281	278	253	269	81	73	80	136	134	144
$C_{55}$	240	240–281	278	253	269	81	73	80	43	48	35
$C_{66}$	263	252–268	283	272	274	1	9	2	122	145	126
$C_{12}$	55	48–71	52	47	49	222	196	213	111	80	109
$C_{13}$	121	118–169	135	105	126	70	63	69	97	70	88
$C_{23}$	121	118–169	135	105	126	70	63	69	99	85	92
$K_{I}$			787	727	753	572	519	555	578	500	569
$K_{II}$			566	544	548	162	146	160	301	290	314
$K_{III}$			566	544	548	162	146	160	272	283	288
$K_{IV}$			556	506	538	2	18	4	244	268	252
$K_{V}$			556	506	538	2	18	4	221	238	226
$K_{VI}$			360	392	349	369	338	354	86	96	70
*B*	220–245	239–260	262	242	250	184	168	178	189	165	187
*G*	225–243	229–243	256	247	248	37	44	37	102	108	100
*E*	502–554	520–555	580	554	560	104	121	105	260	267	256
ν	0.109–0.13	0.137–0.144	0.13	0.118	0.126	0.406	0.38	0.402	0.271	0.231	0.272
G/B	0.99–1.023	0.935–0.958	0.981	1.024	0.995	0.200	0.261	0.21	0.541	0.655	0.539
$\Theta_{D}$	910	921–950	1007	973	971	794	754	779	787	752	774
$H_{v}$	23±0.9	23–56	46	47	45	2	3	2	12	16	12

## Supplementary Materials

The following are available online at https://www.mdpi.com/1996-1944/13/13/3022/s1.

## Abbreviations

The following abbreviations are used in this manuscript:

UHTCs	ultra high-temperature ceramics
DFT	density functional theory
DFPT	density functional perturbation theory
PAW	projector augmented-wave
PP-PW	pseudopotential, plane-wave
XC	exchange-correlation
LDA	local density approximation
GGA	generalized gradient approximation
PBE	Perdew-Burke-Ernzerhof
