# Supplementary material for: New Zirconium Diboride Polymorphs—First-Principles Calculations
Source: Materials (Basel). 2020 Jul 6;13(13):3022. doi: 10.3390/ma13133022 (PMC7372453; doi:10.3390/ma13133022)
Supplement: Supplementary file 1 [file materials-13-03022-s001.pdf]

materials

Supplementary Materials

New Zirconium Diboride Polymorphs: First-Principles Calculations

CIF files:

ZrB2_191_LDA.cif

data_findsym-output

_audit_creation_method FINDSYM

_symmetry_space_group_name_H-M "P 6/m 2/m 2/m"

_symmetry_Int_Tables_number 191

_cell_length_a 3.13453

_cell_length_b 3.13453

_cell_length_c 3.47721

_cell_angle_alpha 90.00000

_cell_angle_beta 90.00000

_cell_angle_gamma 120.00000

loop_

_space_group_symop_id

_space_group_symop_operation_xyz

1 x,y,z

2 x-y,x,z

3 -y,x-y,z

4 -x,-y,z

5 -x+y,-x,z

6 y,-x+y,z

7 x-y,-y,-z

8 x,x-y,-z

9 y,x,-z

10 -x+y,y,-z

11 -x,-x+y,-z

12 -y,-x,-z

13 -x,-y,-z

14 -x+y,-x,-z

15 y,-x+y,-z

16 x,y,-z

17 x-y,x,-z

18 -y,x-y,-z

19 -x+y,y,z

20 -x,-x+y,z

21 -y,-x,z

22 x-y,-y,z

23 x,x-y,z

24 y,x,z

loop_

_atom_site_label

_atom_site_type_symbol

_atom_site_symmetry_multiplicity

_atom_site_Wyckoff_label

_atom_site_fract_x

_atom_site_fract_y

_atom_site_fract_z

_atom_site_occupancy

Zr1 Zr 1 a 0.00000 0.00000 0.00000 1.00000

B1 B 2 d 0.33333 0.66667 0.50000 1.00000

ZrB2_191_PBE.cif

data_findsym-output

_audit_creation_method FINDSYM

_symmetry_space_group_name_H-M "P 6/m 2/m 2/m"

_symmetry_Int_Tables_number 191

_cell_length_a 3.17298

_cell_length_b 3.17298

_cell_length_c 3.52711

_cell_angle_alpha 90.00000

_cell_angle_beta 90.00000

_cell_angle_gamma 120.00000

loop_

_space_group_symop_id

_space_group_symop_operation_xyz

1 x,y,z

2 x-y,x,z

3 -y,x-y,z

4 -x,-y,z

5 -x+y,-x,z

6 y,-x+y,z

7 x-y,-y,-z

8 x,x-y,-z

9 y,x,-z

10 -x+y,y,-z

11 -x,-x+y,-z

12 -y,-x,-z

13 -x,-y,-z

14 -x+y,-x,-z

15 y,-x+y,-z

16 x,y,-z

17 x-y,x,-z

18 -y,x-y,-z

19 -x+y,y,z

20 -x,-x+y,z

21 -y,-x,z

22 x-y,-y,z

23 x,x-y,z

24 y,x,z

loop_

_atom_site_label

_atom_site_type_symbol

_atom_site_symmetry_multiplicity

_atom_site_Wyckoff_label

_atom_site_fract_x

_atom_site_fract_y

_atom_site_fract_z

_atom_site_occupancy

Zr1 Zr 1 a 0.00000 0.00000 0.00000 1.00000

B1 B 2 d 0.33333 0.66667 0.50000 1.00000

ZrB2_191_PBEsol.cif

data_findsym-output

_audit_creation_method FINDSYM

_symmetry_space_group_name_H-M "P 6/m 2/m 2/m"

_symmetry_Int_Tables_number 191

_cell_length_a 3.15616

_cell_length_b 3.15616

_cell_length_c 3.49517

_cell_angle_alpha 90.00000

_cell_angle_beta 90.00000

_cell_angle_gamma 120.00000

loop_

_space_group_symop_id

_space_group_symop_operation_xyz

1 x,y,z

2 x-y,x,z

3 -y,x-y,z

4 -x,-y,z

5 -x+y,-x,z

6 y,-x+y,z

7 x-y,-y,-z

8 x,x-y,-z

9 y,x,-z

10 -x+y,y,-z

11 -x,-x+y,-z

12 -y,-x,-z

13 -x,-y,-z

14 -x+y,-x,-z

15 y,-x+y,-z

16 x,y,-z

17 x-y,x,-z

18 -y,x-y,-z

19 -x+y,y,z

20 -x,-x+y,z

21 -y,-x,z

22 x-y,-y,z

23 x,x-y,z

24 y,x,z

loop_

_atom_site_label

_atom_site_type_symbol

_atom_site_symmetry_multiplicity

_atom_site_Wyckoff_label

_atom_site_fract_x

_atom_site_fract_y

_atom_site_fract_z

_atom_site_occupancy

Zr1 Zr 1 a 0.00000 0.00000 0.00000 1.00000

B1 B 2 d 0.33333 0.66667 0.50000 1.00000

```
# ZrB2_194_LDA.cif

data_findsym-output

_audit_creation_method FINDSYM

_symmetry_space_group_name_H-M "P 63/m 2/m 2/c"

_symmetry_Int_Tables_number 194

_cell_length_a 3.02501

_cell_length_b 3.02501

_cell_length_c 8.51551

_cell_angle_alpha 90.00000

_cell_angle_beta 90.00000

_cell_angle_gamma 120.00000

loop_

_space_group_symop_id

_space_group_symop_operation_xyz

1 x,y,z
```

2 x-y,x,z+1/2

3 -y,x-y,z

4 -x,-y,z+1/2

5 -x+y,-x,z

6 y,-x+y,z+1/2

7 x-y,-y,-z

8 x,x-y,-z+1/2

9 y,x,-z

10 -x+y,y,-z+1/2

11 -x,-x+y,-z

12 -y,-x,-z+1/2

13 -x,-y,-z

14 -x+y,-x,-z+1/2

15 y,-x+y,-z

16 x,y,-z+1/2

17 x-y,x,-z

18 -y,x-y,-z+1/2

19 -x+y,y,z

20 -x,-x+y,z+1/2

21 -y,-x,z

22 x-y,-y,z+1/2

23 x,x-y,z

24 y,x,z+1/2

loop_

_atom_site_label

_atom_site_type_symbol

_atom_site_symmetry_multiplicity

_atom_site_Wyckoff_label

_atom_site_fract_x

_atom_site_fract_y

_atom_site_fract_z

_atom_site_occupancy

B1 B 4 f 0.33333 0.66667 0.02828 1.00000

Zr1 Zr 2 d 0.33333 0.66667 0.75000 1.00000

ZrB2_194_PBE.cif

data_findsym-output

_audit_creation_method FINDSYM

_symmetry_space_group_name_H-M "P 63/m 2/m 2/c"

_symmetry_Int_Tables_number 194

_cell_length_a 3.07656

_cell_length_b 3.07656

_cell_length_c 8.62465

_cell_angle_alpha 90.00000

_cell_angle_beta 90.00000

_cell_angle_gamma 120.00000

loop_

_space_group_symop_id

_space_group_symop_operation_xyz

1 x,y,z

2 x-y,x,z+1/2

3 -y,x-y,z

4 -x,-y,z+1/2

5 -x+y,-x,z

6 y,-x+y,z+1/2

7 x-y,-y,-z

8 x,x-y,-z+1/2

9 y,x,-z

10 -x+y,y,-z+1/2

11 -x,-x+y,-z

12 -y,-x,-z+1/2

13 -x,-y,-z

14 -x+y,-x,-z+1/2

15 y,-x+y,-z

16 x,y,-z+1/2

17 x-y,x,-z

18 -y,x-y,-z+1/2

19 -x+y,y,z

20 -x,-x+y,z+1/2

```
21 -y,-x,z
22 x-y,-y,z+1/2
23 x,x-y,z
24 y,x,z+1/2

loop_
_atom_site_label
_atom_site_type_symbol
_atom_site_symmetry_multiplicity
_atom_site_Wyckoff_label
_atom_site_fract_x
_atom_site_fract_y
_atom_site_fract_z
_atom_site_occupancy
B1 B    4 f 0.33333 0.66667 0.02810 1.00000
Zr1 Zr    2 d 0.33333 0.66667 0.75000 1.00000
```

ZrB2_194_PBEsol.cif

data_findsym-output

_audit_creation_method FINDSYM

_symmetry_space_group_name_H-M "P 63/m 2/m 2/c"

_symmetry_Int_Tables_number 194

_cell_length_a 3.05018

_cell_length_b 3.05018

_cell_length_c 8.56483

_cell_angle_alpha 90.00000

_cell_angle_beta 90.00000

_cell_angle_gamma 120.00000

loop_

_space_group_symop_id

_space_group_symop_operation_xyz

1 x,y,z

2 x-y,x,z+1/2

3 -y,x-y,z

4 -x,-y,z+1/2

5 -x+y,-x,z

6 y,-x+y,z+1/2

7 x-y,-y,-z

8 x,x-y,-z+1/2

9 y,x,-z

10 -x+y,y,-z+1/2

11 -x,-x+y,-z

12 -y,-x,-z+1/2

13 -x,-y,-z

14 -x+y,-x,-z+1/2

15 y,-x+y,-z

16 x,y,-z+1/2

17 x-y,x,-z

18 -y,x-y,-z+1/2

19 -x+y,y,z

20 -x,-x+y,z+1/2

21 -y,-x,z

22 x-y,-y,z+1/2

23 x,x-y,z

24 y,x,z+1/2

loop_

_atom_site_label

_atom_site_type_symbol

_atom_site_symmetry_multiplicity

_atom_site_Wyckoff_label

_atom_site_fract_x

_atom_site_fract_y

_atom_site_fract_z

_atom_site_occupancy

B1 B 4 f 0.33333 0.66667 0.02879 1.00000

Zr1 Zr 2 d 0.33333 0.66667 0.75000 1.00000

ZrB2_59_LDA.cif

data_findsym-output

_audit_creation_method FINDSYM

_symmetry_space_group_name_H-M "P 21/m 21/m 2/n (origin choice 2)"

_symmetry_Int_Tables_number 59

_cell_length_a 3.05717

_cell_length_b 4.93098

_cell_length_c 4.54134

_cell_angle_alpha 90.00000

_cell_angle_beta 90.00000

_cell_angle_gamma 90.00000

loop_

_space_group_symop_id

_space_group_symop_operation_xyz

1 x,y,z

```
2 x+1/2,-y,-z
3 -x,y+1/2,-z
4 -x+1/2,-y+1/2,z
5 -x,-y,-z
6 -x+1/2,y,z
7 x,-y+1/2,z
8 x+1/2,y+1/2,-z

loop_
_atom_site_label
_atom_site_type_symbol
_atom_site_symmetry_multiplicity
_atom_site_Wyckoff_label
_atom_site_fract_x
_atom_site_fract_y
_atom_site_fract_z
_atom_site_occupancy
Zr1 Zr    2 b 0.25000 0.75000 0.38904 1.00000
```

B1 B 4 e 0.25000 -0.06056 -0.08705 1.00000

```
# ZrB2_59_PBE.cif

data_findsym-output
_audit_creation_method FINDSYM

_symmetry_space_group_name_H-M "P 21/m 21/m 2/n (origin choice 2)"
_symmetry_Int_Tables_number 59

_cell_length_a 3.10050
_cell_length_b 5.02883
_cell_length_c 4.60418
_cell_angle_alpha 90.00000
_cell_angle_beta 90.00000
_cell_angle_gamma 90.00000

loop_
_space_group_symop_id
_space_group_symop_operation_xyz
1 x,y,z
```

2 x+1/2,-y,-z

3 -x,y+1/2,-z

4 -x+1/2,-y+1/2,z

5 -x,-y,-z

6 -x+1/2,y,z

7 x,-y+1/2,z

8 x+1/2,y+1/2,-z

loop_

_atom_site_label

_atom_site_type_symbol

_atom_site_symmetry_multiplicity

_atom_site_Wyckoff_label

_atom_site_fract_x

_atom_site_fract_y

_atom_site_fract_z

_atom_site_occupancy

Zr1 Zr 2 b 0.25000 0.75000 0.38562 1.00000

B1 B 4 e 0.25000 -0.06195 -0.08857 1.00000

ZrB2_59_PBEsol.cif

data_findsym-output

_audit_creation_method FINDSYM

_symmetry_space_group_name_H-M "P 21/m 21/m 2/n (origin choice 2)"

_symmetry_Int_Tables_number 59

_cell_length_a 3.07143

_cell_length_b 4.98134

_cell_length_c 4.57772

_cell_angle_alpha 90.00000

_cell_angle_beta 90.00000

_cell_angle_gamma 90.00000

loop_

_space_group_symop_id

_space_group_symop_operation_xyz

1 x,y,z

2 x+1/2,-y,-z

3 -x,y+1/2,-z

4 -x+1/2,-y+1/2,z

5 -x,-y,-z

6 -x+1/2,y,z

7 x,-y+1/2,z

8 x+1/2,y+1/2,-z

loop_

_atom_site_label

_atom_site_type_symbol

_atom_site_symmetry_multiplicity

_atom_site_Wyckoff_label

_atom_site_fract_x

_atom_site_fract_y

_atom_site_fract_z

_atom_site_occupancy

Zr1 Zr 2 b 0.25000 0.75000 0.38692 1.00000

B1 B   4 e 0.25000 -0.06193 -0.08932 1.00000

Phonons:

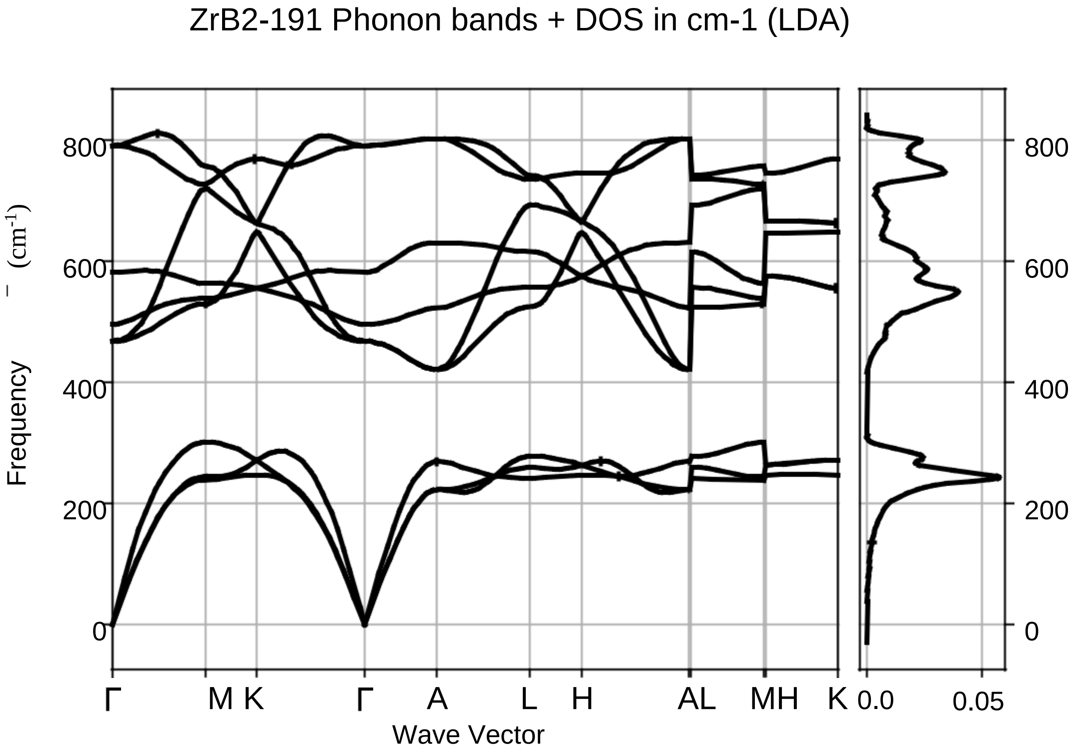
ZrB2-191 Phonon bands + DOS in cm-1 (LDA)
Frequency (cm-1)
800
600
400
200
0
Γ
M K
Γ
A
L
H
AL
MH
K
Wave Vector
0.0
0.05

ZrB$_2$-191 Phonon bands + DOS in cm^{-1} (PBE)

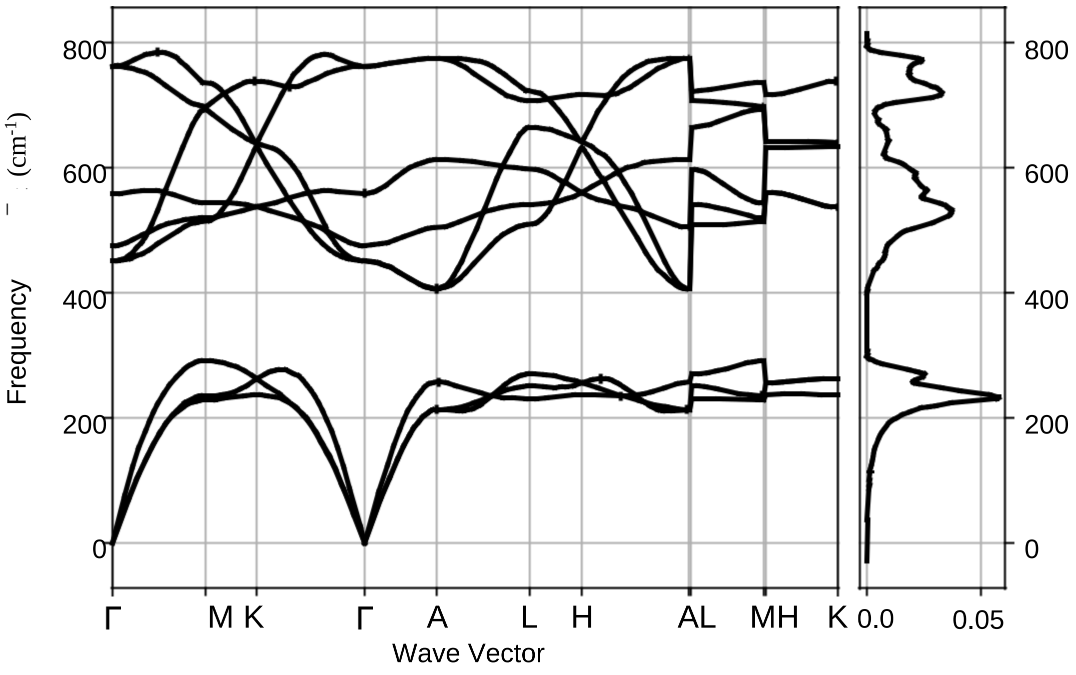

ZrB2-191 Phonon bands + DOS in cm-1 (PBEsol)

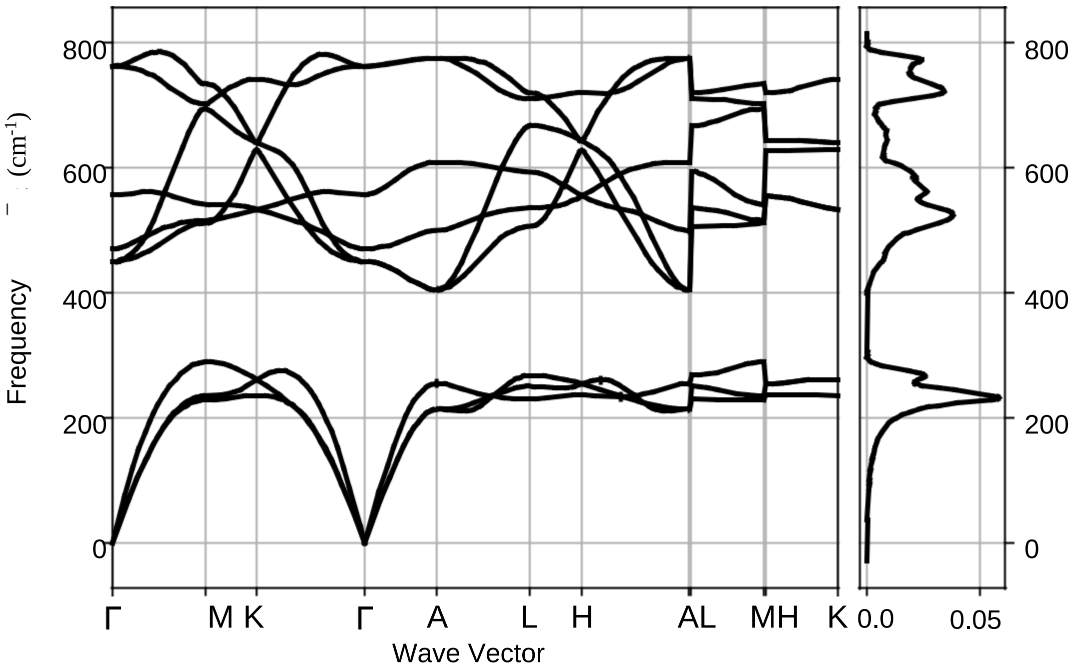

ZrB2-194 Phonon bands + DOS in cm-1 (LDA)

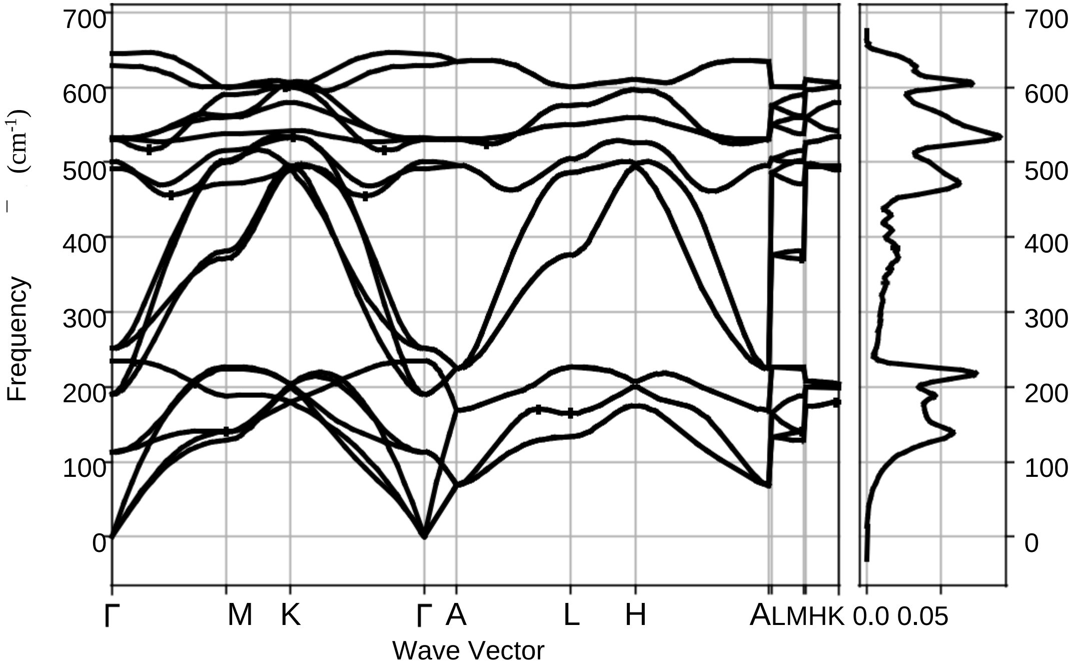

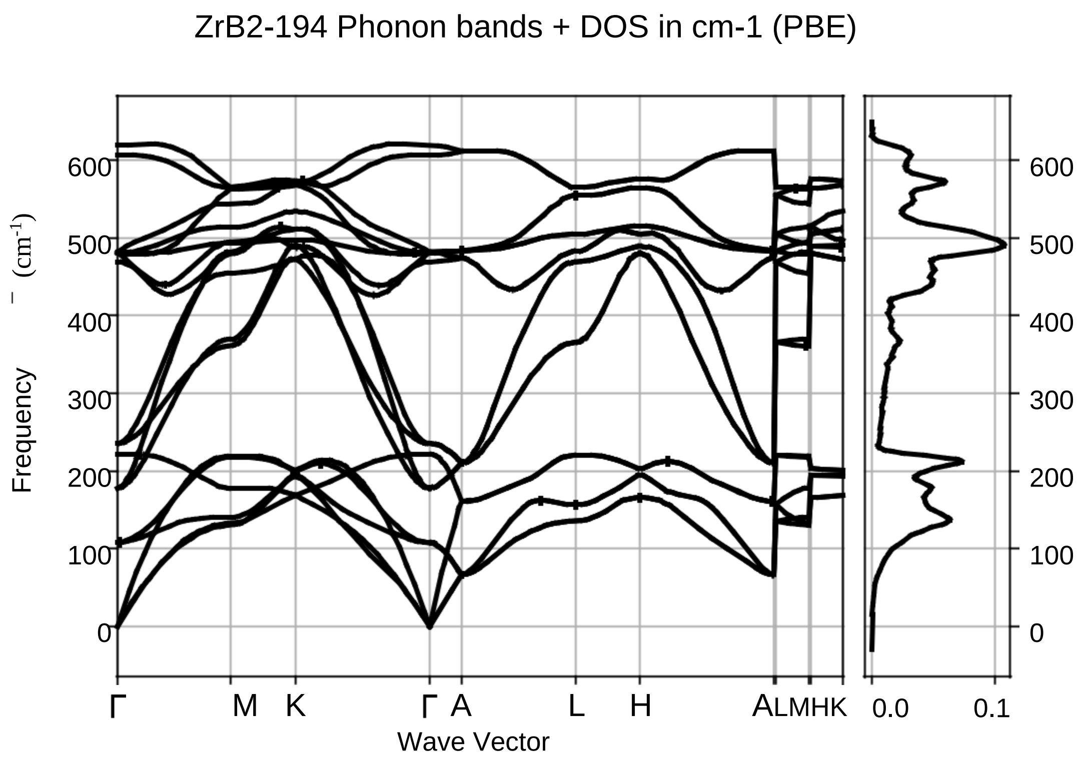
ZrB2-194 Phonon bands + DOS in cm-1 (PBE)
Frequency (cm-1)
600
500
400
300
200
100
0
Γ
M
K
Γ
A
L
H
ALMHK
Wave Vector
0.0
0.1

ZrB2-194 Phonon bands + DOS in cm-1 (PBEsol)

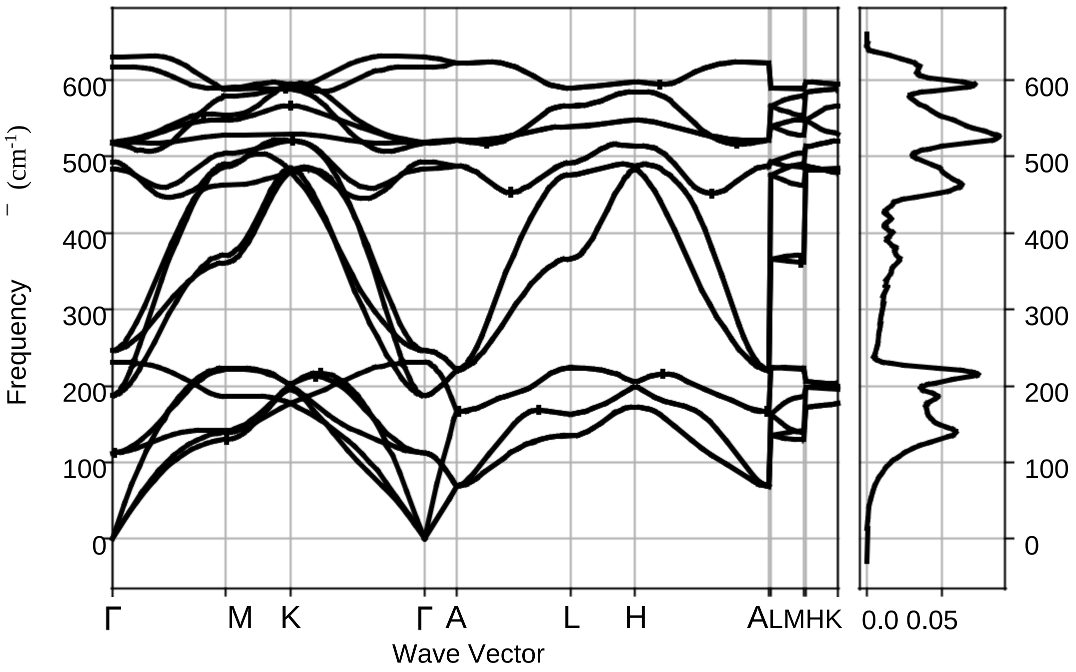

ZrB2-59 Phonon bands + DOS in cm-1 (LDA)

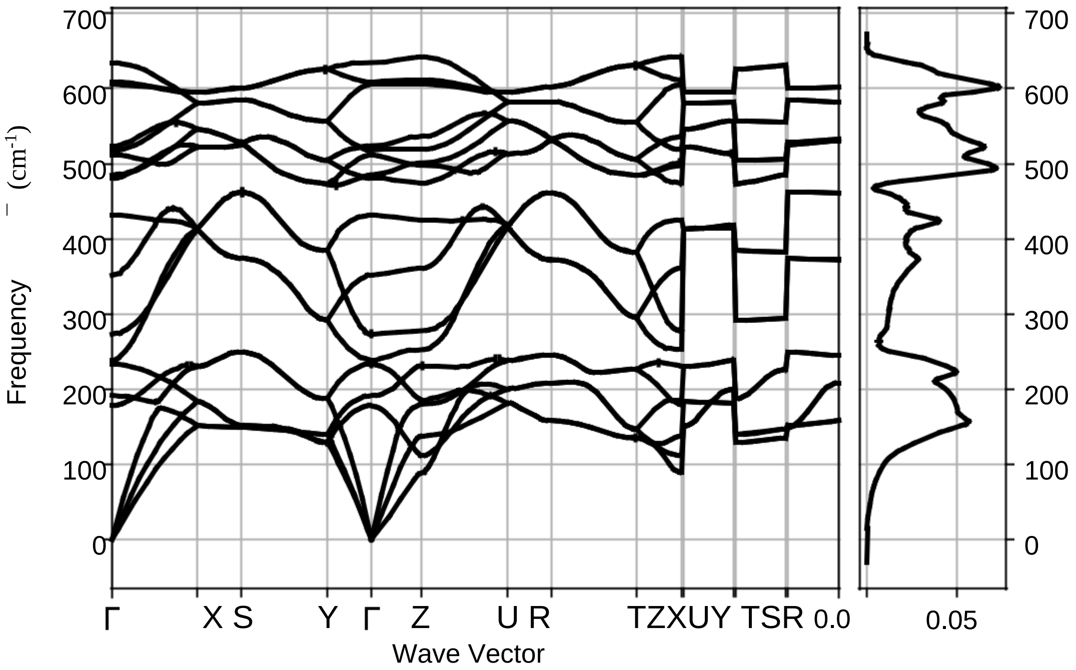

ZrB2-59 Phonon bands + DOS in cm-1 (PBE)

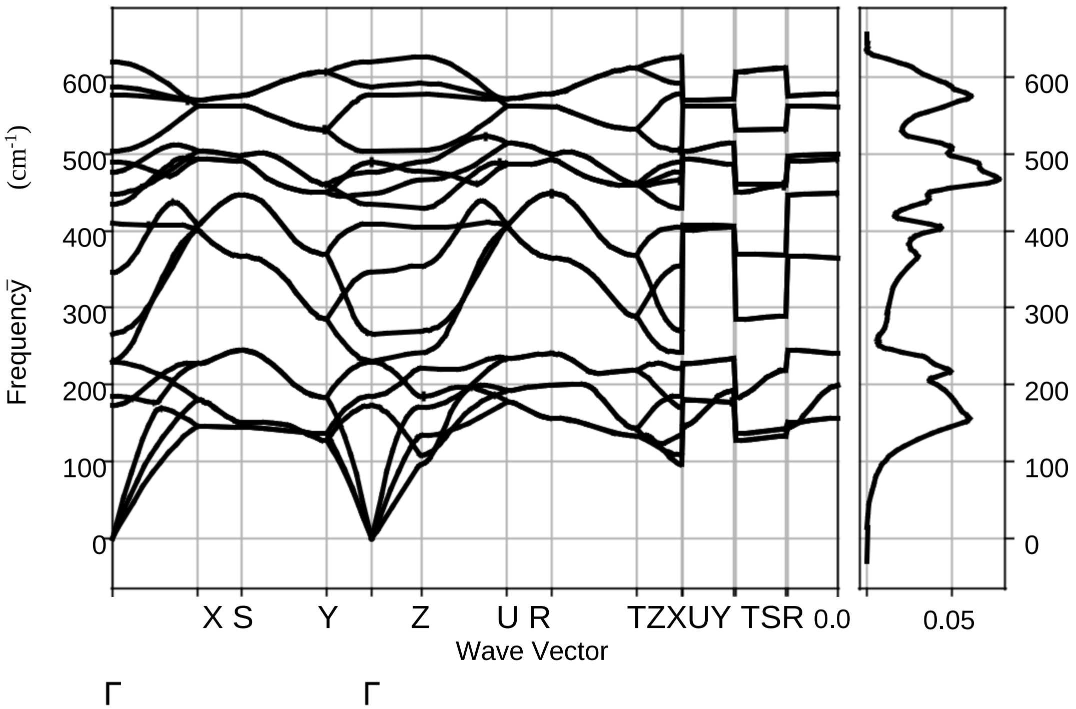

© 2020 by the authors. Licensee MDPI, Basel, Switzerland. This article is an open access article distributed under the terms and conditions of the Creative Commons Attribution (CC BY) license (http://creativecommons.org/licenses/by/4.0/).
